# Flexible Heater Fabrication Using Amino Acid-Based Ink and Laser-Direct Writing

**DOI:** 10.3390/mi13122209

**Published:** 2022-12-13

**Authors:** Sangmo Koo

**Affiliations:** Department of Mechanical Engineering, Incheon National University, Incheon 22012, Republic of Korea; skoo@inu.ac.kr

**Keywords:** bioinspired system, tryptophan, photoreduction, laser-direct writing, nanoparticle, sintering

## Abstract

Nature’s systems have evolved over a long period to operate efficiently, and this provides hints for metal nanoparticle synthesis, including the enhancement, efficient generation, and transport of electrons toward metal ions for nanoparticle synthesis. The organic material-based ink composed of the natural materials used in this study requires low laser power for sintering compared to conventional nanoparticle ink sintering. This suggests applicability in various and sophisticated pattern fabrication applications without incurring substrate damage. An efficient electron transfer mechanism between amino acids (e.g., tryptophan) enables silver patterning on flexible polymer substrates (e.g., PET) by laser-direct writing. The reduction of silver ions to nanoparticles was induced and sintered by simultaneous photo/thermalchemical reactions on substrates. Furthermore, it was possible to fabricate a stable, transparent, and flexible heater that operates under mechanical deformation.

## 1. Introduction

Metal nanoparticles can be synthesized through diverse methods, such as laser ablation [1], photoreduction [2], and chemical reduction [3], which use polymer surfactants, reducing agents, and compound reductions. Although there is rapid growth in the extensive applications of nanotechnologies, on the contrary, significant concerns about environmental impacts have emerged regarding toxicity during nanomaterial-based processes [4,5]. For example, hazardous materials are used and generate toxic materials during the development process as a postprocessing step. In order to overcome this limitation, interest in ecofriendly nanoparticle synthesis, which is inspired by real biosystems in nature, is increasing [6,7,8,9]. Research on nanomaterial synthesis using a wide range of nature-derived materials and microorganisms (e.g., fungus, algae, viruses, yeast, and bacteria) has been conducted [10,11,12,13,14,15]. It is mainly advantageous due to its easy availability, use of nontoxic chemicals, environmental friendliness, cost-effectiveness, and relative reproducibility. For example, chemical/physical methods for silver (Ag) nanoparticle synthesis using a nontoxic method in an aquatic environment have been proposed [16,17,18].

The sintering of nanomaterials (e.g., nanoparticles and nanowires) using a laser has been proposed as a new approach for metal pattern fabrication [17,19,20,21]. In conventional annealing, the transfer of thermal energy to the substrate, as well as to the target material, is inevitable [22,23]. The required temperature for annealing reaches up to several hundreds of degrees Celsius. During this process, the excessive thermal energy remains and is transferred to the thermally vulnerable substrate (e.g., the polymer), resulting in severe deformation. Therefore, the mismatch between the thermal vulnerability of flexible substrates, which cannot withstand the required amount of thermal energy, is a challenge to be solved in a flexible electrode fabrication. The strength of laser-based sintering is the controllability of thermal energy and low-temperature processes. Furthermore, the melting temperature of the nanomaterials can be reduced from 200 to 100 °C due to the thermodynamic size effect [24]. By applying marginal thermal energy to nanomaterials through the optimization of the laser irradiation parameters, it is possible to fabricate an electrode pattern on the heat-sensitive substrates.

In particular, silver nanoparticles have a lower thermal conductivity than bulk silver. Therefore, silver is an appropriate material for sintering on a substrate that is easily deformed by heat. Moreover, the mechanical stability of the fabricated silver pattern has also been shown. Therefore, silver patterns are applied to various applications, such as sensors and touch panels [25].

When preparing a precursor for nanoparticle synthesis in an ecofriendly manner, the choice of solvent medium, environmentally friendly reducing agents, and nontoxic stabilizing agents for the stabilization of the nanoparticles should be considered.

For nanoparticle synthesis, various types of organic solvents have been used, and water is the most nontoxic solvent [26]. In this study, water was used as a solvent for the precursor preparation for silver nanoparticle synthesis.

Next, amino acids, which are the essential components of proteins, can be used as ecofriendly reducing agents for nanoparticle synthesis. Amino acids play a critical role in the body’s metabolism, and amino acids are uniquely modified during the chemical reaction. The hydrocarbon moiety remains; however, the -NH_2_ and -COOH groups undergo chemical transformation. This chemical transformation induces the imbalance, generation, and transfer of electrons [17,27,28,29]. Therefore, it was noted that there could be a possibility of synthesizing nanoparticles from metal ions using this mechanism. Tryptophan (Trp), an aromatic amino acid, was used as a reducing agent among amino acids in this study. It plays a physiological role, functioning both independently or through polymerization into larger molecules. Trp is used for the synthesis of niacin (vitamin B_3_), which is a precursor of serotonin. Trp is susceptible to light and is transformed into Trp indolyl radicals and the subsequently generated electrons. Additionally, p-p stacking occurs, and electron transport (e.g., proton-coupled electron transfer) is accelerated due to the chemical structure of tryptophan [30]. For example, the generated electrons (through the proton-coupled electron transfer process) of tryptophan are capable of transferring between tryptophan residues in the apoenzyme of deoxyribonucleic acid (DNA) photolyze. The electrons can also be transferred between excited flavin adenine dinucleotide (FADH) and Trp [31]. The subsequent three successive electron transfers occur in three Trp residues. Similarly, it is able to synthesize metal nanoparticles by the photoreduction of metal ions through the election generation and transfer of Trp.

Finally, it is necessary to consider the stabilizer of the synthesized nanoparticles. The stabilizing agent protects the particles and prevents particle agglomeration. Among the substances, gelatin is suitable as a capping and stabilizing agent. Gelatin is a natural polymer extracted from collagen by partial hydrolysis and shows excellent biocompatibility [32]. Therefore, it has been used in diverse applications, such as wound healing and drug carriers [33,34]. This gelatin prevents the aggregation and over-growth of synthesized nanoparticles [32,35]. As a result, it enables uniform nanoparticle generation and stable maintenance within the intermolecular gelatin structure. Moreover, residual gelatin can be removed only with DI water, which is carried out easily and without using toxic solvents for development. Furthermore, gelatin is also homogeneous and optically transparent. A uniform coating can also be achieved using an optimal concentration. Therefore, gelatin is a suitable material for laser-based fabrication.

In this study, a highly precise electroconductive metal pattern can be fabricated through an efficient electron transfer mechanism using the aromatic amino acid (tryptophan) in an ecofriendly manner. Besides, the excellent mechanical/electrical properties of the electrode were confirmed through various performance tests (e.g., repeated bending), and it was possible to fabricate a heater with excellent performance by adopting this pattern.

## 2. Materials and Methods

### 2.1. Preparation of Precursor Material and Characterization

A precursor was prepared for silver nanoparticle synthesis by a photoreduction process. It was composed of a mixture of silver nitrate (AgNO_3_, Sigma Aldrich, St. Louis, MO, USA), tryptophan (Sigma Aldrich, St. Louis, MO, USA), and gelatin from porcine skin (gel strength 300, type-A, Sigma Aldrich, St. Louis, MO, USA), and this was dissolved in deionized (DI) water. The Trp solution was prepared by mixing 0.25 g Trp and 10 mL DI water vigorously for 2 h using a magnetic stirrer at room temperature. Then, this solution was filtered using a syringe filter (0.2-micron, Sigma Aldrich, St. Louis, MO, USA). To minimize additional reactions with light, the prepared tryptophan aqueous solution was stored in a brown bottle. The gelatin solution was prepared by dissolving about 2 g of gelatin in 8 mL of 60 °C DI water using a hot plate stirrer (Daihan sci, Wonju, Republic of Korea) for 2 h. The tryptophan and 5 mL 1 M silver nitrate solutions were mixed with the gelatin solution using a hot plate stirrer (Daihan sci, Wonju, Republic of Korea) for 30 min to prepare a uniform precursor. The prepared precursors were also stored in a brown bottle to prevent reactions with external light in a vacuum chamber. For the characterization of the precursor, the absorption spectra were measured using a UV–Vis spectrophotometer (J-5100B, Beijing, China) (Figure S2). The nanoparticles were characterized using a field emission scanning transmission electron microscope (TEM, Talos F200X, Thermo Fisher Scientific, Waltham, MA, USA) operated at 200 kV, which provided 0.25 nm point resolution. The nanoparticle sizes were analyzed by TEM image using ImageJ (NIH, Bethesda, MD, USA) and MATLAB (Mathworks, Natick, MA, USA) for image processing. High-resolution X-ray diffraction patterns of the nanoparticles were collected using a diffractometer (HR-XRD, SmartLab, Rigaku, Tokyo, Japan) with a CuKα radiation source (λ = 1.5412 Å) of 45 kV and 200 mA. The measurement carried out in reflection geometry and the diffraction (Bragg) angles 2θ were scanned at a step of 0.025.

### 2.2. Laser Setup & Pattern Fabrication

**Figure 1:**
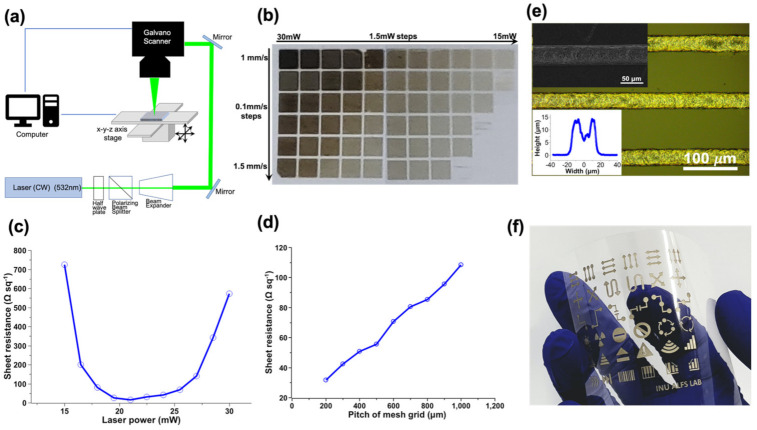
Laser-based fabrication. (**a**) Schematic of laser system for sintering; (**b**) image of patterning for parametric study, according to scanning speed and laser power; (**c**) change in sheet resistance by changing the laser power at a constant scanning speed of 1.2 mm/s; (**d**) change in sheet resistance with different pitch of the grid pattern with 22.5 mW of laser power and 1.2 mm/s of scanning speed; (**e**) image of the sintered pattern on PET (inset: (top): SEM image and inset (bottom): cross-sectional profile of a sintered pattern), and (**f**) the diverse shape pattern on the PET substrate.

Before spin coating, the polyethylene terephthalate (PET, Sigma Aldrich, St. Louis, MO, USA) substrate was cleaned with isopropyl alcohol (Sigma Aldrich, St. Louis, MO, USA) in an ultrasonic bath for 10-min, respectively, then dried in a vacuum oven (Daihan sci, Wonju, Republic of Korea) at 80 °C. Before dropping the precursor material, we applied the oxygen plasma for the enhancement of wettability. To obtain a thin and uniform film, the precursor was spin-coated using a spin coater for 30 s at 1000 rpm. The spin-coated film was mounted on an X-Y-Z stage (Boardtech, Incheon, Republic of Korea). The laser beam was aligned perpendicular to the stage. A pattern was fabricated using a continuous laser (Finesse pure, Laser quantum, wavelength = 532 nm, maximum power = 3 W). The laser power was adjusted with an attenuator comprised of a half-wave plate (WPH10M-532, Thorlabs, Newton, NJ, USA) and polarizing beam splitter (PBS, Thorlabs, Newton, NJ, USA). A galvanometer scanner (HurrySCAN II, Scanlab, Munchen, Germany) with an f-theta lens (S4LFT5165/292, SILL Optics, Wendelstein, Germany) was adopted for arbitrary shape pattern fabrication (Figure 1a), and its spot size was approximately 750 μm. The mirror position of the galvanometer scanner was controlled using software (SAMLight, SCAPS, Oberhaching, Germany). Laser power was measured using a power meter (S121C, Thorlabs, Newton, NJ, USA) at the inlet of the galvanometer scanner. To prevent unwanted reactions due to external light, the patterning was performed in a dark room. After the fabrication process, the sample was immersed in warm DI water for 30 s and air-dried by blowing N_2_ gas. The sample was stored in a vacuum chamber to minimize the oxidation of samples during long-term storage. To check whether the generated nanoparticles (using the precursor) could be used for patterning, the resistivity was calculated. The single electrode was fabricated on the glass substrate, and the resistance was measured. The silver paste was applied at each end of the electrodes to reduce the contact resistance between the pattern and the probe tips. The resistivity was calculated using $\rho =R\frac{l}{A}$, where R is resistance, A is the cross-sectional area, and l is the length of the single electrode.

### 2.3. Pattern Characterization

To characterize the cross-section topography of a pattern, a surface profiler (DEKTAK XT-E, Bruker, Billerica, MA, USA) was used. The shape of the pattern was carried out using scanning electron microscopy (SEM, JEOL JSM-780F, Tokyo, Japan). To verify the connectivity of the pattern, the resistance was measured using a two-probe technique using a semiconductor parameter analyzer (HP4155A, Hewlett-Packard, Palo Alto, CA, USA). To reduce the contact resistance between the pattern and the probe tips, silver paste was applied at both ends of the electrodes, and the Sheet resistance was calculated using a function of $R_{s}=R\frac{w}{L}$, where R is the resistance, w is width, and L is the length of the mesh-type panel.

### 2.4. Heater Fabrication and Temperature Measurement

The transparent heater was fabricated with a mesh-type pattern with 800 μm spacing on PET using optimal fabrication conditions (22.5 mW of laser power, 1.2 mm/s of scanning speed). The silver paste was applied to both ends of the meshed pattern, and the copper tape was attached to the silver paste. The experiment was conducted after the silver paste was sufficiently dried. The DC voltage was supplied to the heater, and temperature rise by the generated heat was measured using an infrared (IR) camera (T630sc, FLIR, Wilsonville, OR, USA).

## 3. Results and Discussion

**Figure 2:**
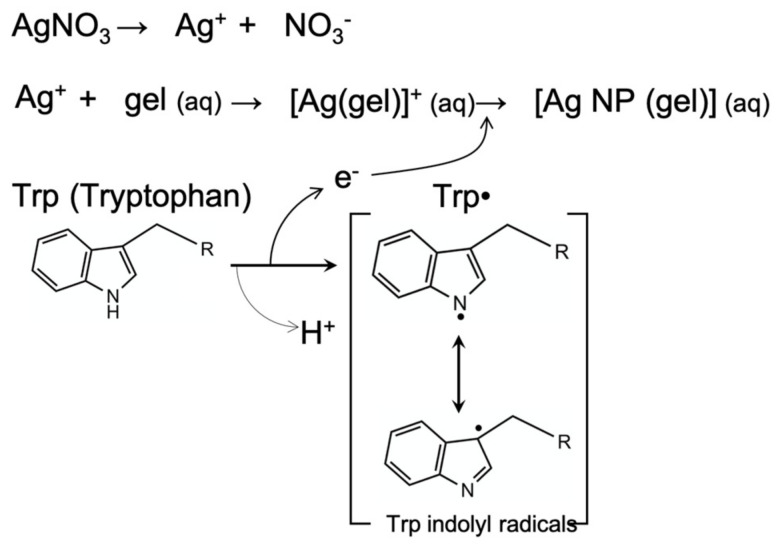
Main schematic of silver nanoparticle generation by photoreduction.

It was possible to synthesize organic-based precursor ink using the efficient electron transfer of tryptophan for silver nanoparticle synthesis and sintering. When a laser is irradiated into the precursor, the photoreduction of silver ions into nanoparticles and the sintering of the generated nanoparticles occurs, subsequently, in one step (Figure 2). Consideration of the optimal laser fabrication parameters (e.g., the coating thickness of the precursor ink, laser power, scan speed, etc.) is required to fabricate the electrode pattern on the PET substrate without damage. The pattern fabricated using optimal conditions showed stable operation under mechanical deformations, such as bending and twisting. These electrodes were applied to the heater fabrication, and the stable operation of a flexible heater was observed under various conditions (e.g., voltage change and long-term operation).

### 3.1. Optimal Concentration of the Constituent Precursor for Laser-Based Sintering

For precursor preparation, the Ag ion concentration was fixed as 1M for uniformity in this research, and the optimal concentration of the other constituent substances (gelatin and tryptophan) was studied by changing the weight % of these materials compared to the Ag ion solution.

First, gelatin acts as a matrix or stabilizer in nanoparticle synthesis and also affects the coating thickness. Polymers enable the synthesis and maintenance of stable nanoparticles by minimizing oxidation, aggregation, and precipitation through the formation of nanoparticle complexes [36,37]. Gelatin plays a similar role due to its thermoreversible properties [38]. As the temperature rises, the gelatin polypeptide chains change into the form of flexible, unrolled coils. However, it is partially reconstituted into a 𝛼-helix that is aligned through the self-assembly of 3D polypeptides as the temperature drops. During this process, microscale domains are created inside the gelatin and form a stable gelatinous complex (${\left[Ag\left(gel\right)\right]}^{+}\left(aq\right)$) with the silver ions. The silver nanoparticles can coexist in the microdomain of gelatin for a long time. Furthermore, gelatin acts as a template and controls the size and uniformity of the synthesized nanoparticles [39]. Next, the gelatin concentration also affects the precursor coating thickness, which is related to the laser fabrication results, such as pattern shape and adhesion [40]. Therefore, optimal gelatin concentration should be considered for efficient patterning on a polymer substrate, such as PET. The coating can be damaged by ablation, resulting in an incomplete pattern using thin precursor coatings. In contrast, in the patterning with a thick coating, when the energy of the irradiated laser is insufficient, the electrode does not attach to the substrate. In this case, the pattern is washed off during the postprocessing process. Therefore, the adhesion and coating thickness have a trade-off relationship. The precursor thickness also affects the width of the resulting electrode. Longer irradiation time and higher laser power are required to allow the pattern to adhere to the substrate with a thicker coating. Thermal conduction in the film layer occurs in the vertical and lateral directions, simultaneously. Therefore, thermal conduction in the lateral direction also increases with a thick coating, resulting in a wider pattern.

The precursor coating thickness can be controlled by changing the gelatin concentration in an aqueous solution. When the gelatin concentration is lower than 3 wt%, it is not properly available for the coating on the substrate due to low viscosity. However, if the gelatin concentration is higher than 18 wt%, rapid gelation occurs during the spin coating process at room temperature, resulting in nonuniform coating. The film coating thickness changed from 3.7 μm to 19.8 μm by changing the gelatin concentration from 3 wt% to 18 wt%. In this study, a precursor with a gelatin concentration of 15 wt% was used, and its coating thickness was 18.8 μm.

Next, we considered the Trp concentration, which is used as a reducing agent. The appropriate concentration of tryptophan in the precursor is also an important factor in nanoparticle synthesis and patterning. As the laser irradiated the tryptophan, the tryptophan would be oxidized and emit one electron during the photolysis process [41].

The generation of nanoparticles could be checked through the solution color change (Figure S1). The darker the solution, the higher the synthesis of the nanoparticles. When the Trp concentration in the precursor was lower than 10 mM, the generation of nanoparticles was significantly low. However, the degree of nanoparticle synthesis remained constant in Trp concentrations above 30 mM. Therefore, precursors containing 30 mM tryptophan were used in this study, and the synthesis of the silver nanoparticles was also characterized using UV–Vis absorption spectra with a wavelength ranging from 250 to 700 nm (Figure S2).

The sintered film was prepared using optimal laser processing conditions (22.5 mW of laser power, 1.2 mm/s of scanning speed) after spin coating at 2000 rpm for 15 s for X-ray diffraction (XRD) measurement. The sintered film was dried in the vacuum chamber for 10 min at room temperature before XRD measurement to remove the residual solvent after washing. The XRD data showed sharp peaks at 2θ = 38.2°, 44.5°, 64.56°, and 77.54°, which corresponded to the (111), (200), (220), and (311) planes. This confirms that the silver sheet had a face-centered cubic (FCC) crystalline structure (Figure S3).

The generation of the nanoparticles used the precursor with optimal gelatin and Trp concentrations, and their size was measured using transmission electron microscopy (TEM). The diameter of the nanoparticles was in the range of 14–26 nm (Figure S4).

In this study, a precursor was prepared by mixing silver ions, gelatin (as a stabilizing agent), and tryptophan (as a reducing agent) for ecofriendly nanoparticle synthesis. The generated electrons via the photolysis of tryptophan induced the reduction of the silver ions into nanoparticles, even without harsh chemical conditions (e.g., the use of sodium hydroxide). Gelatin stabilizes the generated nanoparticle. The optimal constituent concentration of the precursor not only synthesizes silver nanoparticles but also coats the precursor film for successful laser-based pattern fabrication.

### 3.2. Laser-Based Pattern Fabrication

#### 3.2.1. Optimal Condition for Sintering on PET

The advantage of laser-based sintering is that it can pattern even on thermally vulnerable polymer substrates. The laser can be intensively focused only on a restricted area, and it is possible to synthesize and sinter the nanoparticle efficiently. Therefore, it was also able to minimize the denaturation of the polymer substrate. Commonly used polymer substrates include materials such as polydimethylsiloxane (PDMS) [17], poly(methyl methacrylate) (PMMA) [42], polyethylene terephthalate (PET) [19,22], polyethylene naphthalate (PEN) [43], and polyimide (PI) [44]. In this study, PET was selected as the polymer substrate.

Next, the parameters for laser processing were considered for efficient laser patterning. For optimal patterning on a polymer substrate, the following factors need to be considered: (i) maintenance of the adhesion between the pattern and the substrate, and (ii) the minimization of substrate damage. The patterning on the polymer substrate through laser-based sintering showed a unique mechanism, unlike other substrate materials (e.g., glass). As the laser irradiated, the area affected by thermal energy was limited only to the highly localized region, and this is referred to as the heat-affected zone (HAZ). At this region, the melting of the substrate and the immersion of the sintered pattern occurs within a short time, and the subsequent solidification allows the pattern to adhere to the melted region through mechanical interlocking [45]. Therefore, finding the required marginal energy, which is needed for melting the substrate, the sintering of the nanoparticle, and the immersion and adhesion of the sintered pattern to the substrate, is required.

The maximum temperature rise at the surface can be theoretically described for relatively slower laser scan speeds and surface absorptions as

$$\Delta T_{\max}\left(Z^{*}=0\right)≃\frac{\theta_{c}}{2\sqrt{\pi }h_{s}^{*}}\ln\left(\frac{16}{{\xi v}_{s}^{*}{}^{2}}\right)$$

where $\theta_{c}$, $h_{s}^{*}$, and $v_{s}^{*}$ correspond to the normalized laser power, the film thickness, and the scanning speed, respectively. ξ is a constant and it defined as $\exp\left(C+1\right),$ where C is 0.577 [46]. Therefore, the maximum temperature rise at the surface is affected by the laser power, coating thickness, and scan speed of the laser. In order to find the optimal conditions, such as laser power and scan speed, which can generate the restricted HAZ and minimize substrate deformation and pattern damage, a parametric study was performed.

The optimal energy density and scanning speed required for the patterning was obtained by changing the energy power (from 15 mW to 30 mW, with 1.5 mW steps) and the scanning speed (from 1 mm/s to 1.5 mm/s, with 0.1 mm/s steps) (Figure 1b). The adhesion between the pattern and the substrate was weak with low laser power and high scanning speed. When there is a low laser power and a low scan speed with the same energy density, the synthesis and sintering of the nanoparticles were not achieved efficiently, resulting in incomplete patterns. These patterns tend to lift off during the cleaning process with DI water. Under the conditions of an energy density greater than approximately 3 J/cm^2^ and a scanning speed of less than 1.1 mm/s, the pattern and substrate are damaged at the same time. In contrast, when the energy density is less than 1.6 J/cm^2^, and the scanning speed is more than 1.4 mm/s, the pattern tends not to adhere well to the substrate, and its connectivity is poor.

The quality and applicability of the patterns were tested using the sheet resistance (R_s_) of 20 samples. The sheet resistance, according to the pitch of the patterns (25 mm × 25 mm mesh-type), was measured using two terminal methods. When there is excessive energy (laser density up to 2.8 J/cm^2^), which exceeds the required energy for sintering, this results in a sudden drop in electrical conductivity. In contrast, a sudden drop in the electrical conductivity was also observed for the incomplete pattern, which was fabricated using insufficient energy (laser density below 1.6 J/cm^2^) (Figure 1c). Sheet resistance tends to increase with increasing pattern spacing (Figure 1d). From the parametric study, the optimal fabrication condition was 2.5 J/cm^2^ for energy density and 1.2 mm/s for scan speed. The highest electrical conductivity was observed for the fabricated pattern using optimal conditions. The lowest resistivity was about 372 nΩ m, which is 23.4 times higher than bulk Ag (15.9 nΩ m at 20 °C).

This value was judged to be suitable for a microscale electrode, and this optimal fabrication condition was used for the experiment. The pattern, which is a fabricated pattern using the optimal conditions, was homogeneous, with a width of about 50 μm and a height of 1.5 μm (Figure 1e). In this study, a galvanic scanner was adopted. The galvanic scanner-based sintering method has the advantage of the processability of a variety of complex patterns in the restricted region. This represents the possibility of being used in diverse applications (Figure 1f).

#### 3.2.2. Attachment Stability of the Pattern

The stable adhesion of the pattern on the substrate was also tested to check stable functioning under an external physical loading condition. In order to test this, a tape pull and an ultrasonic test were performed.

The tape-pull test was performed using commercial adhesive tape (Scotch^®^ MagicTM tape, 3M). The tape was attached to the sintered 1.5 cm × 1 cm pad, with applied pressure, and peeled off over 10 times. Pattern damage or separation from the substrate was not observed. This was confirmed by observing whether the separated/detached pattern remained on the adhesive side or not (Figure S5a). Next, an adhesion test was also performed by applying ultrasonic waves for 30 s in an ultrasonic bath. The pattern was stably maintained on the substrate without separation (Figure S5b).

As a result, the stable adhesion of the pattern on a substrate was observed without damage under pressured and ultrasonic conditions.

#### 3.2.3. Mechanical Flexibility Test of the Pattern

Mechanical flexibility and electrical reliability tests were performed under bending and twisting.

**Figure 3:**
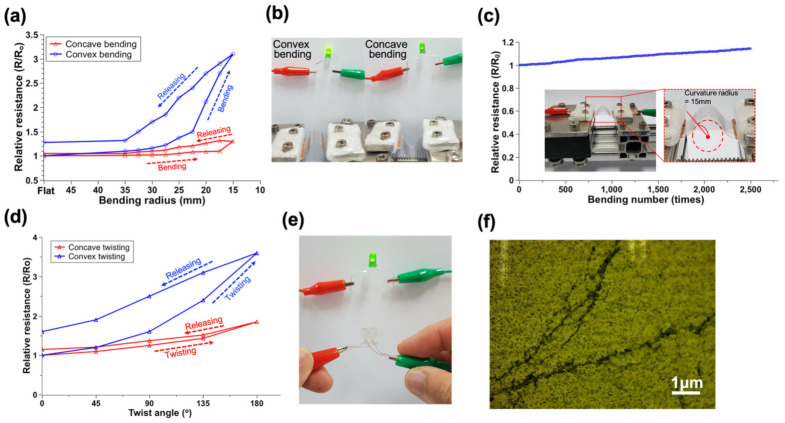
Electrical performance test of the meshed-type pattern on PET. (**a**) Relative resistance change with different bending radius; (**b**) image of convex and concave bending on the circuit; (**c**) relative resistance change from repeated bending with a bending radius of 15 mm; (**d**) relative resistance change with different twisting angles; (**e**) image of twisted pattern on PET, and (**f**) an optical microscope image of microscale crack after repeated bending test.

First, various bending conditions were applied to the pattern to check the stable operation of the pattern under bending. Resistance was measured at both ends of the pattern to check for pattern damage during the bending test. Fifty patterns with a length of 30 mm were fabricated on a PET, and silver paste was applied to both ends. The copper tape was attached to the silver paste to minimize the contact resistance effect. The bending radius of the substrate was set from 35 mm to 15 mm by adjusting the interval of the mechanical stage, and the change in the relative resistance (R/Ro) to the bending radius was measured (Figure 3a). The relative resistance tends to increase with a decreasing curvature radius for the bent substrate. The pattern’s stability under different bending directions (e.g., convex and concave) of the substrate was also tested. Because the pattern is subjected to opposite loads (tensile and compression) to the bending direction (Figure 3b), when the substrate is bent in the concave direction, the patterns are subjected to a compressive load; however, the patterns are subjected to a tensile load when the substrate is bent in the convex direction. In the concave mode, the relative resistance was negligible up to a bending radius of 20 mm; however, the relative resistance increased by up to 1.32 as the bending radius decreased to 17.5 mm. When the substrate returned to an initial (flat) state, the relative resistance was maintained and increased by about 5%. However, the relative resistance increased to a maximum of 3.1 when the bending radius was changed to 15 mm in the convex mode. The relative resistance was maintained and increased by about 28% when the substrate returned to an initial state. The tendency of resistance to increase in convex-mode bending was more noticeable than that in concave-mode bending (Figure 3a).

Next, the pattern stability under repeated bending was tested on the mechanical stage (Figure 3c). By fixing one end of the pattern and moving the other end repeatedly with a constant frequency (0.5 Hz) and curvature radius (15 mm), the resistance change was measured at the original (flat) state after every cycle of 10 bends. During 2500 bending cycles, the resistance increased by about 14.3% of the original value (Figure 3c). This is predicted to be a result of pattern damage caused by repeated bending and the contact resistance between the sintered pattern and the copper electrode. In order to visualize pattern connectivity during the bending test, an electrical circuit with a light-emitting diode (LED) was constructed, and significant LED flickering was not observed during the repeated bending test. However, the relative resistance increased significantly, and the LED flickered after about 4500 times of bending. This is expected to result from a disconnection in the pattern from cyclic loading.

Furthermore, another pattern stability test under mechanical deformation twisting was measured using relative electrical conductivity, similar to the bending test. The twisting angle changed from 0 to 180 degrees (Figure 3d). As one side is fixed and the other side is twisted in opposite directions (pattern/substate direction), the patterns are subjected to different types of loads (tensile/compression). As the twist angle increased in the concave mode, which puts it under a compressive load, the relative resistance increased by about 1.85. When returning to the initial state, the relative resistance was maintained at about a 15% increase. However, the relative resistance increased by up to 3.6 in the convex mode twisting, which is subjected to tension loading. The relative resistance was maintained at about a 60% increase when returning to the flat state. An electrical circuit with a light-emitting diode (LED) was constructed to visualize the deformation and connectivity of the patterned electrode during the twisting test process. Significant blinking of the LED was not observed during the twisting (Figure 3e).

For both bending and twisting, an increase in resistance was observed as the patterns were deformed and returned to their original state. This tendency is attributed to microscale crack generation during the deformation (Figure 3f). This phenomenon is more evident in the deformation that applied tension.

In this section, the stable maintenance of the pattern’s shape and electrical properties under external physical deformation (e.g., bending and twisting) were tested. It represents the applicability of the material to diverse applications subjected to various types of physical loads.

#### 3.2.4. Transparency Tests of the Pattern

The transparency change of the pattern was also measured. It was possible to fabricate a panel with high permeability and electrical conductivity by patterning a meshed electrode on transparent PET by adjusting the pattern’s spacing transmittance. Transmittance was measured by changing the wavelength from 400 nm to 800 nm for each meshed pattern with different spacings (from 600 to 1000 μm). Constant transmittance in this wavelength range was observed with each spacing of the pattern. However, transmittance tends to decrease with decreasing pattern spacing. (Figure S6a). Based on the data for sheet resistance change according to the pitch of the meshed pattern obtained in the previous section, the relationship between sheet resistance and transmittance was plotted for a specific wavelength (550 nm). The sheet resistance and the transmittance increased at the same time as the increase in the pattern’s spacing occurred (Figure S6b).

### 3.3. Heater Fabrication

**Figure 4:**
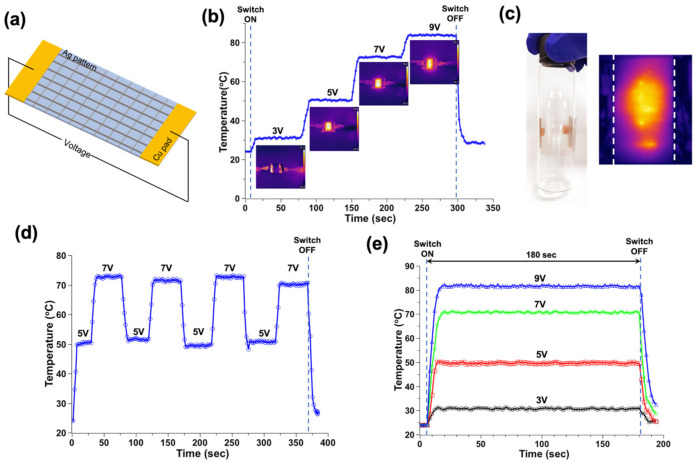
Heater fabrication. (**a**) Schematic of the fabricated heater; (**b**) response of heater from DC voltage (3, 5, 7, and 9V) change and infrared image; (**c**) infrared image of the heater under the bent heater on glass vial (diameter = 25.9 mm); (**d**) response of heater under repetitive DC voltage (5 and 7V) change, and (**e**) long-term operation of the heater under a constant DC voltage (3, 5, 7, and 9V) for 180 s.

The transparent heater was fabricated with a mesh-type pattern with 800 μm spacings on PET. Heat was generated after the DC voltage was supplied to the heater, and temperature rise was measured using an infrared (IR) camera. The temperature reached 32.2, 49.5, 72.2, and 82 ℃ when a constant DC voltage was applied stepwise from 3 to 9V at 2V intervals to the heater for 1 min, respectively. The heater showed a fast temperature response and convergence to the plateau according to voltage change (Figure 4b). In order to test stable heater operation in the bending condition, the heater was attached to the glass vial (20 mL, diameter: 25.9 mm) surface, and temperature distribution was measured. When a 7 V DC voltage was applied, the maximum temperature was 68.9 °C, and stable operation was observed (Figure 4c). In order to test the stable performance of the heater under repetitive voltage changes, repetitive DC voltage (5 and 7 V) was applied at 1 min intervals for 30 s. The convergence temperature was about 52 °C for 5 V, and 72 °C for the 7 V DC voltage condition. Even after repeated experiments, significant losses of function were not observed.

For the test of long-term stable heater operation, a constant DC voltage (3, 5, 7, and 9V) was supplied for 3 min. The constant temperature (32.2, 49.5, 72.2, and 82 °C) was observed with the constant DC voltage (3, 5, 7, and 9V), respectively.

As a result, a heater with a fast response and stable long-term operation could be fabricated through laser-based sintering using tryptophan-based ink.

## 4. Conclusions

Interest in the environmentally friendly synthesis and fabrication techniques of nanoparticles is increasing. A precursor, which was composed of silver ions, gelatin, and an amino acid (tryptophan) with higher electron transfer properties, was synthesized in this study. The silver nanoparticles were synthesized through the efficient photoreduction of silver ions. These synthesized nanoparticles sintered, enabling fast metal patterning on a flexible polymer (PET) substrate. Therefore, an electrode pattern with high transmittance and electrical conductivity was made possible; it also showed high structural stability against external physical deformation (e.g., bending, twisting). Based on these results, a high-performance transparent/flexible transparent heater could be fabricated. This heater showed a fast response time and long-term stable functionality under changes in DC voltage. In conclusion, through the combination of tryptophan-based ink synthesis and laser-based sintering, an efficient, ecofriendly metal patterning method is reported in this study. This can be expected to expand to various applications.

## Data Availability

The data presented in this study are available in this article.

## Supplementary Materials

The following supporting information can be downloaded at: https://www.mdpi.com/article/10.3390/mi13122209/s1, Figure S1. Generation of silver nanoparticles by laser irradiation (a) Image of laser irradiation on the precursor (upper) and silver nanoparticle synthesis (lower) and (b) Image of nanoparticle generation after laser irradiation for 1 h with 5 mW of laser power; Figure S2. UV-Vis absorption spectra of precursor to characterize the silver nanoparticle synthesis; Figure S3. X-ray diffraction (XRD) data for silver patterning fabricated using Trp-based precursor; Figure S4. Transmission electron microscope (TEM) results (a) Image of TEM and (b) Particle size distribution of synthesized silver nanoparticles; Figure S5. Adhesion test for mechanical stability between the pattern and substrate. (a) Image of tape-pull test, and (b) ultrasonication test of pattern on PET substrate; Figure S6. Transmittance spectra of meshed pattern (a) Transmittance result with different pitches of pattern, and (b) Relation between transmittance and sheet resistance at a constant wavelength (550 nm) and different pitches. References [47,48,49,50,51] are cited in the Supplementary Materials.
