# Supplementary material for: Flexible Heater Fabrication Using Amino Acid-Based Ink and Laser-Direct Writing
Source: Micromachines (Basel). 2022 Dec 13;13(12):2209. doi: 10.3390/mi13122209 (PMC9783287; doi:10.3390/mi13122209)
Supplement: Supplementary file 1 [file micromachines-13-02209-s001.zip › micromachines-2033124-supplementary.pdf]

micromachines

Supplementary Materials

Flexible Heater Fabrication using Amino acid-based Ink and Laser-Direct Writing

Sangmo Koo*

1. Generation of silver nanoparticles by laser irradiation

As the laser was irradiated to the precursor material, it was observed the color change of precursor to brown (Figure S1 (a)). When the laser was irradiated for 1 hour with 5 mW of laser power, the color of the precursor solution changed clearly (Figure S1(b)).

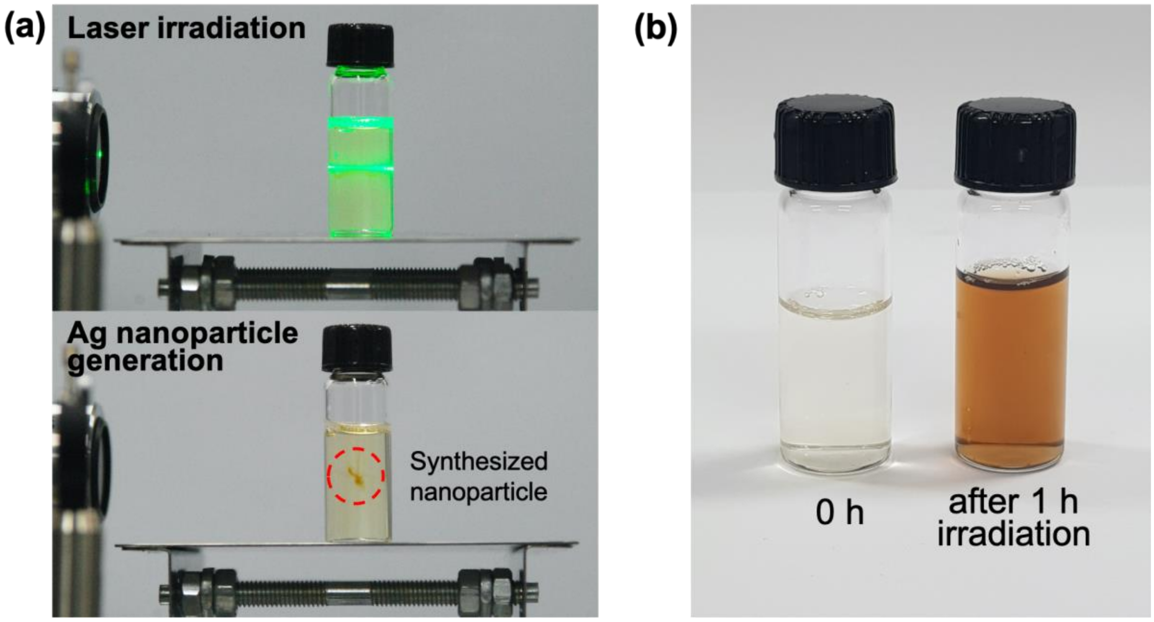

Figure S1. Generation of silver nanoparticles by laser irradiation (a) Image of laser irradiation on the precursor (upper) and silver nanoparticle synthesis (lower) and (b) Image of nanoparticle generation after laser irradiation for 1h with 5mW of laser power.

2. Characterization of silver nanoparticle synthesis (UV-Vis absorption spectra)

The synthesis of silver nanoparticles was characterized using UV–Vis absorption spectra (wavelength ranging from 250 to 700 nm) (Figure S2). It shows the absorption spectrum of UV-visible light in the laser-irradiated sample with time. The non-laser irradiated sample was selected as a reference sample. The silver nanoparticles have free electrons that yield a surface plasmon resonance (SPR) absorption band. This is due to the mutual vibration of the electron of silver nanoparticle in resonance with a light wave [1,2]. The shape of the peak represents the surface plasmon resonance of the silver nanoparticles. Moreover, the increase in the intensity of the plasmon band can be predicted by a decrease in the bandwidth [3]. From the experimental results, it was observed that the wavelength of the surface plasmon peak point generated at 440 nm, and decreased as the concentration increased. When irradiated for a long time (6 hours), in a high-concentration solution, it was also observed that the wavelength of the peak gradually shifted to 433 nm. This shift may be due to a blue shift which is dependent on particle size and shape [4,5]. Therefore, it can be confirmed that efficient silver nanoparticle synthesis with the increase of the laser irradiation time on the precursor.

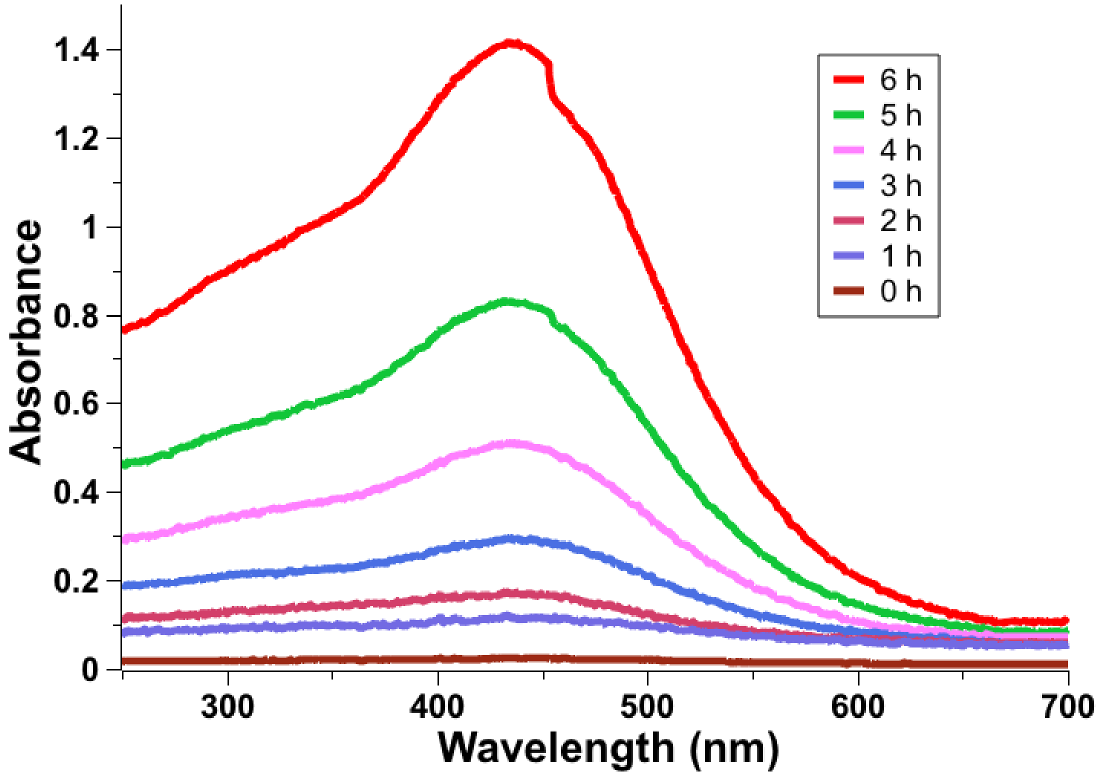

Figure S2. UV-Vis absorption spectra of precursor to characterize the silver nanoparticle synthesis

3. Characterization of synthesized silver nanoparticle (XRD)

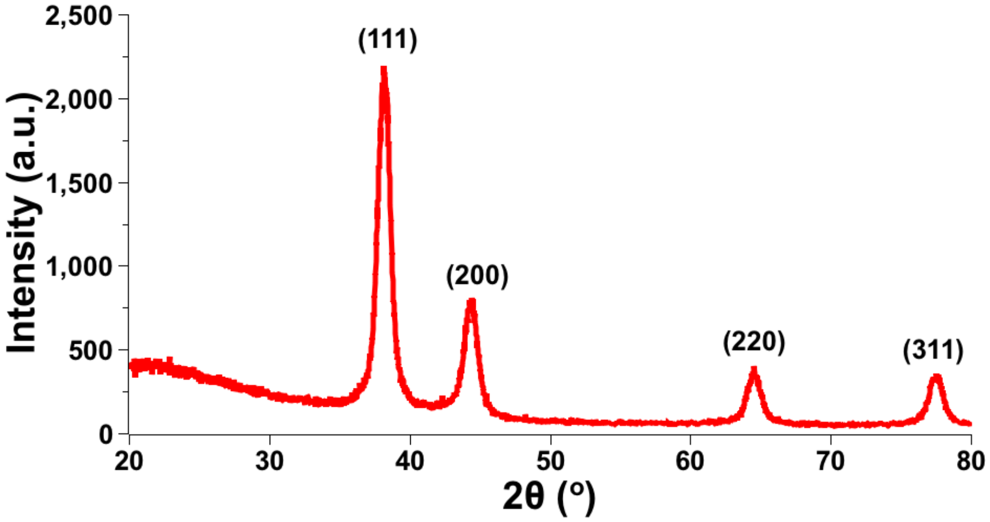

Figure S3. X-ray diffraction (XRD) data for silver patterning fabricated using Trp-based precursor

4. Characterization of synthesized silver nanoparticle (TEM)

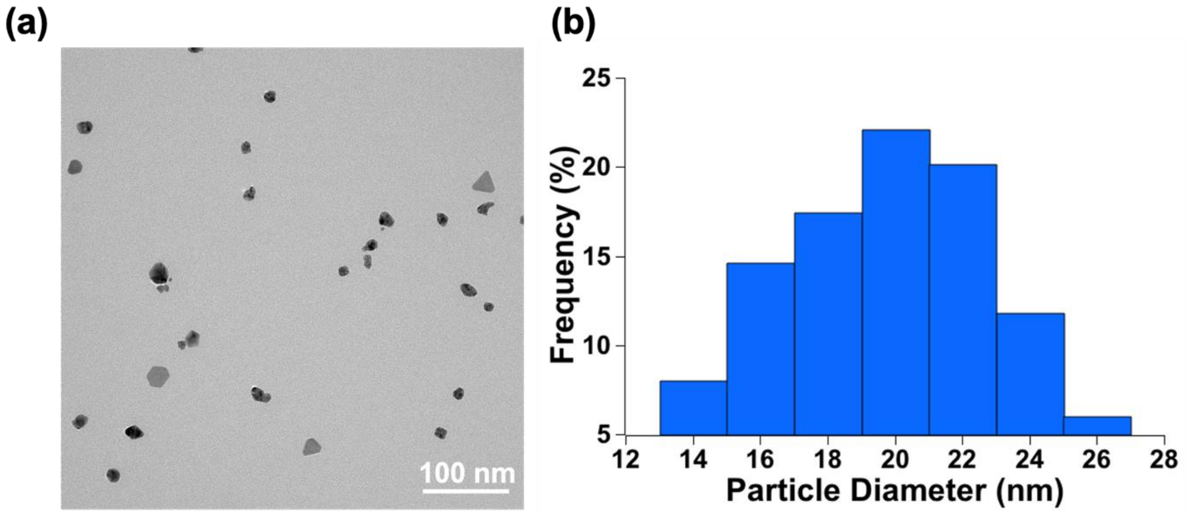

Figure S4. Transmission electron microscope (TEM) results (a) Image of TEM and (b) Particle size distribution of synthesized silver nanoparticles

5. Adhesion test for mechanical

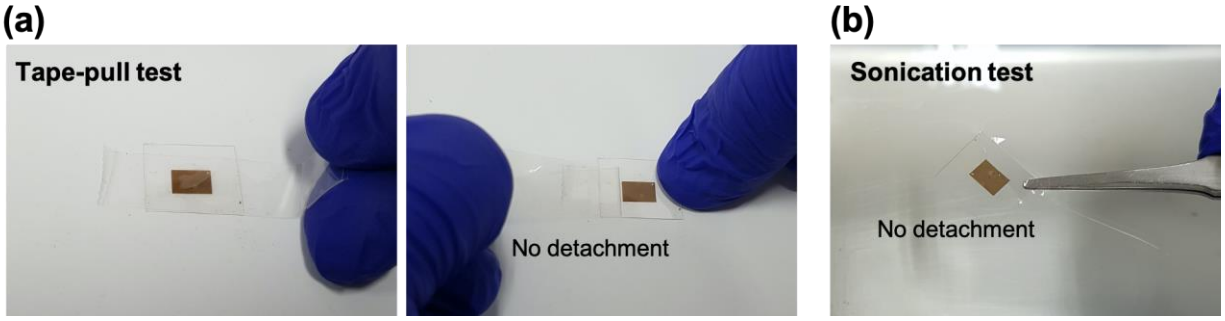

Figure S5. Adhesion test for mechanical stability between the pattern and substrate. (a) Image of tape-pull test, and (b) ultrasonication test of pattern on PET substrate.

6. Transmittance spectra of meshed pattern on PET

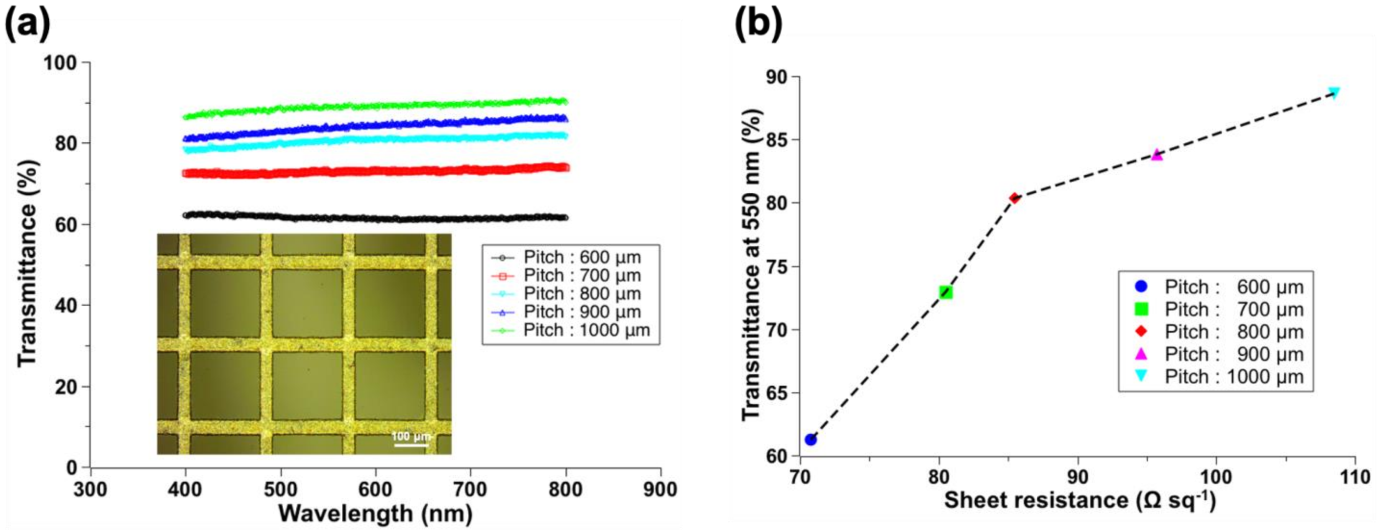

Figure S6. Transmittance spectra of meshed pattern (a) Transmittance result with different pitches of pattern, and (b) Relation between transmittance and sheet resistance at constant wavelength (550 nm) and different pitches.

Reference

1. Anandalakshmi, K.; Venugobal, J.; Ramasamy, V. Characterization of silver nanoparticles by green synthesis metho d using Pedalium murex leaf extract and their antibacterial activity. *Applied Nanoscience (Switzerland)* **2016**, *6*, 399-408, doi:10.1007/s13204-015-0449-z.
2. Njagi, E.C.; Huang, H.; Stafford, L.; Genuino, H.; Galindo, H.M.; Collins, J.B.; Hoag, G.E.; Suib, S.L. Biosynthesis of Iron and Silver Nanoparticles at Room Temperature Using Aqueous Sorghum Bran Extracts. **2010**, 10.1021/la103 190n, doi:10.1021/la103190n.
3. Zhang, W.; Qiao, X.; Chen, J. Synthesis and characterization of silver nanoparticles in AOT microemulsion system. *Chemical Physics* **2006**, *330*, 495-500, doi:10.1016/j.chemphys.2006.09.029.
4. Kelly, K.L.; Coronado, E.; Lin Lin Zhao, a.; Schatz, G.C. The Optical Properties of Metal Nanoparticles: The Influ ence of Size, Shape, and Dielectric Environment. **2002**, 10.1021/jp026731y, doi:10.1021/jp026731y.
5. and, K.-S.L.; El-Sayed, M.A. *Gold and Silver Nanoparticles in Sensing and Imaging: Sensitivity of Plasmon Response to Size, Shape, and Metal Composition*; American Chemical Society: 2006; 10.1021/jp062536y.
